# Correlation of single-fiber electromyography studies and functional status in patients with amyotrophic lateral sclerosis

**DOI:** 10.1515/med-2024-0990

**Published:** 2024-06-29

**Authors:** Róbert Rostás, István Fekete, László Horváth, Sándor Márton, Klára Fekete

**Affiliations:** Division of Radiology and Imaging Science, Department of Medical Imaging, Faculty of Medicine, University of Debrecen, 4032 Debrecen, Hungary; Department of Neurology, Faculty of Medicine, University of Debrecen, 4032 Debrecen, Hungary; Department of Pharmaceutical Surveillance and Economy, Faculty of Pharmacy, University of Debrecen, 4032 Debrecen, Hungary; Institute of Political Science and Sociology, Faculty of Humanities, University of Debrecen, Debrecen, Hungary

**Keywords:** amyotrophic lateral sclerosis, fiber density, jitter, orbicularis oculi muscle, frontalis muscle

## Abstract

**Objective:**

Our aim was to examine the significance of single-fiber electromyography (SFEMG) in patients diagnosed with amyotrophic lateral sclerosis (ALS) and determine the best correlating parameter with SFEMG parameters and clinical scales across different muscles including facial muscles.

**Methods:**

SFEMG examinations were conducted on the extensor digitorum (ED), frontalis, and orbicularis oculi muscles. Mean jitter, percentage of increased jitter, fiber density (FD), and impulse blocking percentage were compared to reference values and functional scales.

**Results:**

Significant differences (*p* < 0.001) were observed between the patients’ SFEMG results and reference values in all muscles. Significant correlations were found between SFEMG parameters and clinical scales, particularly when considering both FD and jitter. A notable value of the ALS Functional Rating Scale Revised (ALSFRS-R) was detected in all muscles: 31 points in the ED muscle, 30 in the orbicularis oculi muscle, and 31 in the frontalis muscle. Below this ALSFRS-R threshold, the percentage of increased jitter was higher, while FD remained relatively low.

**Conclusion:**

SFEMG examination emerges as a valuable tool for better understanding ALS and holds potential for assessing prognosis. Combined jitter and FD analysis showed the strongest correlation with clinical scales. In addition to the ED muscle, the orbicularis oculi muscle may be important in the assessment.

## 1 Introduction

Amyotrophic lateral sclerosis (ALS) is the most common incurable motor neuron disease (MND) in adults, which is characterized by dysfunction of the upper motor neuron (UMN) and the lower motor neuron (LMN) resulting in progressive weakness and wasting of muscles in the bulbar, limb, thoracic, and abdominal regions [1]. In a recent publication, according to data from a meta-analysis, the standardized global incidence of ALS was 1.68 per 100,000 person-years of follow-up. Nevertheless, it may vary in different regions: in Europe and North America, it ranges between 1.71 and 1.89 per 100,000, in Asian populations 0.73 and 0.94 per 100,000, and a much higher figure, 2.25 per 100,000 was found in Oceania [2]. ALS phenotypes seem to be similar in different populations, even across Europe according to ALS registries these might be different [3]. The demographic projections indicate that the number of affected individuals aged above 60 years is going to increase and as a consequence of this, the global count of patients grappling with MND, notably ALS, is anticipated to witness an upward trajectory [1,4,5,6,7]. ALS is also known to emerge differently in different regions; it might also have subtle differences, as different registries in Europe pointed out. In ALS, the process of denervation involves a breakdown in communication between motor neurons and the muscle fibers they typically innervate. Subsequently, surviving motor neurons attempt to compensate for this disruption by establishing new connections known as reinnervation [3]. Based on previous literature, the single-fiber electromyography (SFEMG) examination may serve as a suitable tool for detecting these neuropathological abnormalities [8].

The clinical diagnosis of ALS requires the presence of progressive degeneration of UMN or LMN throughout the neuraxis. Electrodiagnostic evaluation is essential in the early stages of ALS, especially when UMN or, more frequently, LMN signs may be hidden [9]. Neurophysiological diagnostic tools play a substantial role in confirming the diagnosis of ALS. Nevertheless, more detailed examinations might be necessary in the early stages of the disease when symptoms first manifest in the patient. Peripheral nerve conduction studies (NCS) and electromyography (EMG) are appropriate diagnostic tools that confirm key pathological features of ALS, as outlined in the El Escorial criteria [10,11], and help differential diagnostics [9,10,11]. Other neurophysiological tests are available to examine the integrity of the nervous system, including SFEMG [12].

SFEMG has demonstrated its diagnostic value in neuromuscular disorders, particularly in cases of myasthenia gravis, and is the most sensitive technique for detecting neuromuscular transmission defects [8,12]. The process of denervation–reinnervation is the pathological hallmark of the ALS [3,9]. Denervation involves the disconnection between motor neurons and muscle fibers they normally control. In response to such disconnection, surviving motor neurons try to compensate by forming new connections, referred to as reinnervation. The presence of abnormal jitter, fiber density (FD), and impulse blocking show a positive correlation with weakness and muscle atrophy [13]. This is likely due to the existence of immature sprouting of nerve terminals and the degree of reinnervation [14].

Classification according to the presenting symptoms can be done, and bulbar-onset, limb-onset (upper and lower), pure upper limb onset, and pure lower limb onset subtypes are usually distinguished [3].

After diagnosing a patient with ALS, the first question from the patient and family is usually associated with the time of survival. Although the progression of ALS can differ considerably, the average duration from symptom onset is approximately 3 years, but it can vary from 2 to 10 or even 20 years [14]. Survival may be influenced by several factors like clinical symptoms at onset, rate of progression, early presence of respiratory failure, nutritional status of the patient, etc. By following our patients more closely, some factors (e.g., nutritional status, respiratory failure) might be treated or their severity reduced [3]. One of the primary reasons is that recognizing the disease in a timely manner and estimating the prognosis allow for the opportunity of appropriate treatment and adequate support, which could improve both quality of life and disease outcomes [15]. Additionally, early diagnosis assists patients and their families in accessing the necessary therapies, support, and resources.

Estimating the prognosis may be crucial, not only from a social aspect but also from the aspect of treatment and clinical trials as well [3,16]. Besides, functional scales like ALS Functional Rating Scale Revised (ALSFRS-R) [17] other measurable methods might be useful [18,19].

The objective of this study was to examine the alterations and clinical significance of SFEMG in patients diagnosed with ALS in different muscles and find the best correlating parameter with clinical scales. Contrary to limb muscles, facial muscles tend to be relatively spared during the early stages of the disease, so our aim was also to find alterations of SFEMG parameters in facial muscles as well. We hypothesize that SFEMG in ALS might give supportive information and better insight into the pathomechanism of the disease and we assume that the findings in the facial muscles are important in this.

## 2 Materials and methods

### 2.1 Patients and data

The study was conducted at the Department of Neurology, University of Debrecen, over an inclusion period from June 1, 2018, to June 30, 2022. The neurophysiology unit, serving as a tertiary center, covers a catchment area of 600,000 residents within a 90 km radius.

In this study, patients meeting the Awaji criteria following the initial diagnosis of ALS were enrolled [10,11]. Each patient exhibited progressive motor impairment within 1 year of diagnosis. Notably, all patients displayed clinical signs of UMN and LMN dysfunction in at least one body region, as confirmed by repeated clinical assessments. The definitive diagnosis of ALS and voluntary participation in the examinations constituted the inclusion criteria. Additionally, confirmation of LMN dysfunction was established through EMG in all enrolled patients. Cases of other types of MNDs, as well as those who did not give consent, were considered exclusion criteria.

To exclude other potential causes of neuropathies that may mimic ALS, motor and sensory NCS were carried out in all patients. To rule out any structural abnormalities of the brain or spinal cord that may potentially mimic ALS, all patients in our study underwent cranial and cervical high-resolution 3-T magnetic resonance tomography. Additionally, cerebrospinal fluid and blood tests were conducted in all cases to exclude the presence of other diseases.

Considering the initial symptoms, the patients were categorized into the following subgroups: bulbar-onset and limb-onset. As ALS symptoms are typically asymmetrical, we created additional subgroups within the limb-onset category to compare our data. These subgroups included patients whose right upper limb (RUL), left upper limb (LUL), right lower limb (RLL), and left lower limb (LLL) were affected. By chance the patients reported themselves to be right handed. So, we did not create dominant and non-dominant groups.

ALSFRS-R score [17] and the Medical Research Council (MRC) strength scale for the extensor digitorum (ED) muscle [18,19] were used to assess functional status and compare the subgroups.

With ALSFRS-R scoring 12 physical functions, in 4 domains are tested: speech, salivation, swallowing, handwriting, nutrition and the use of the hand when eating, dressing and hygiene, turning in bed, walking, climbing stairs, dyspnoea, orthopnoea, respiratory insufficiency. Each item is scored on a scale between 0 and 4, where a score of 4 signifies unimpaired functionality, while a score of 0 denotes severe impairment. The scores are then summed up to obtain a total score that ranges between 0 and 48. Elevated scores correlate with heightened levels of functional self-sufficiency, whereas diminished scores correlate with more pronounced deteriorations in functional capacity [17,20,21]. MRC scale is a frequently employed method to evaluate muscle strength, ranging from Grade 5 (normal) to Grade 0 (absence of visible contraction) [18,19].

### 2.2 SFEMG

Volitional SFEMG was performed using a disposable SF needle electrode with a recording diameter of 25 μm. During the examination, the subjects were in a relaxed state, either comfortably reclining in a chair or lying down in a supine position. The examination was conducted in a quiet, electrically shielded, and temperature-controlled room in the neurophysiology laboratory at the Department of Neurology. The single-fiber EMG test was evaluated using a 9031A006401 Keypoint Clinical System (Natus Medical, Pleasanton, CA, USA). A disposable SFEMG needle electrode (Spes MEdica, Genova, Italy 0.45 (26G) × 37 mm) was used in all cases. Prior to the SFEMG testing, neither the healthy control individuals nor the ALS patients received any medication that could potentially affect the results of the SFEMG study.

Selective recordings of single muscle fiber potentials were performed in the ED muscle in all patients to obtain measurements of mean jitter, percentage of increased jitters, FD, and percentage of impulse blocking. ED was chosen because it was clinically intact at the time of the examination. Clinically detectable unequivocal LMN signs were detected in the RUL group, this is why we showed the analysis also with the exclusion of the RUL group. No denervation, fasciculation, or polyphasic waves were present in the ED muscle. Since we have done voluntary SFEMG we needed to choose muscles that the patient could innervate and maintain constant force for this purpose ED muscle was suitable in all patients. Jitter was calculated on the basis of the mean consecutive difference, while blocks were determined by the count of dropped discharges during consecutive discharges. The percentage of increased jitter and percentage of blocking was calculated from the 20 analyzed potential pairs (numbers of pathological findings/20). FD was defined as the number of single musclefibers from one motor unit within the electrode’s uptake area. Since the facial muscles tend to be relatively spared – at least at the early stages of the course of ALS – we also performed SFEMG examinations of the right frontalis muscle in eight cases and the right orbicularis oculi muscle in seven cases. The limited number of participants may be due to the fact that the needling test involving facial muscles is considered more uncomfortable than similar assessments involving skeletal muscles. Consequently, part of the patient population refused to participate in the frontal and orbicularis oculi.

For jitter analysis, we used age-adjusted healthy controls (*N* = 26) (without any diseases affecting the neuromuscular system), according to reference values used [22].

The electrode position was delicately adjusted to optimize the action potential (AP) amplitudes; for accurate jitter measurements, all relevant APs should exhibit sharply rising phases and sufficient amplitudes.

Twenty potential pairs of APs were measured and recorded from various parts of the muscle, utilizing three to four skin insertions.

During an SFEMG examination, APs greater than 200 µV are inferred to originate from muscle fibers within 300 µm of the recording surface. By measuring the mean number of time-locked APs with an amplitude of greater than 200 µV and a rise time of less than 300 µs at multiple sites, we can calculate FD, which quantifies the local concentration of muscle fibers within the motor unit [23,24].

FD measurements were made by observing the signals on the screen. As the patient voluntarily activated the muscle, the electrode was positioned to record the AP with maximum amplitude from one muscle fiber. This AP triggers an oscilloscope sweep and is delayed for display, allowing the counting of synchronized APs with amplitudes over 200 µV [23,24].

APs are recorded at 20 separate sites within a muscle, minimum at 3 separate insertion sites. FD represents the mean number of APs, including the triggering AP, counted in the 20 sites.

### 2.3 Statistical analysis

We used the SPSS for Windows 19.0 program suite (SPSS Inc., Chicago, USA). for statistical analyses. In this study, we employed a multivariate analysis of variance test and pairwise comparison to examine the interplay between age, gender, and duration, as well as the ALSFRS-R and ED strength score of MRC within distinct ALS subgroups. We compared the data obtained during the SFEMG examination with the data in the normal range [21] using the Mann–Whitney test. In cases where a normal distribution was observed, we applied the Student’s *t*-test. Furthermore, we assessed the correlation between the results of the functional tests and the SFEMG indices using the Spearman’s rank test. Statistical significance was considered if *p* < 0.05.


**Informed consent:** All of the participants signed an informed consent to take part in the protocol, which was approved by the local ethics committee (RKEB5036-2018).
**Ethical statement:** The study was approved by the Regional and Institutional Ethics Committee of University of Debrecen Clinical Centre (protocol number: RKEB5036-2018).

## 3 Results

### 3.1 Baseline characteristics

The study included a cohort of 26 patients who were enrolled based on meeting the Awaji criteria following the initial diagnosis of ALS [10,11]. Of the 26 patients enrolled, 12 were females (aged 45–78, mean age 62 years) and 14 males (aged 49–84 years, mean age 60 years). Healthy controls (*N* = 26) were age and gender (12 females, 14 males) matched.

Patients’ data on disease duration, ALSFRS-R score, and MRC strength score in the ED muscle concerning bulbar and limb grouping are summarized in Table 1. Table 2 contains further details of the limb-onset groups. We did not find any statistically significant differences among the subgroups in terms of gender distribution, and duration of the disease (Tables 1 and 2). In the bulbar group, patients were more than 10 years younger on average (Tables 1 and 2).

**Table 1:** Patient characteristics in different subgroups among the examined ALS patients

	Bulbar-onset	Limb-onset	*p*
Number of patients	8	18	—
Number of female/male	5/3	7/11	0.27
Age (years) (mean ± SD)	57.5 ± 10.0	68.0 ± 11	**0.015**
Disease duration (*from onset to referral, examination*) (month) (mean ± SD)	6.5 ± 1.2	5.94 ± 1.51	0.42
(minimum; maximum)	(6; 8)	(4; 8)	
ALSFRS-R (mean ± SD)	30.4 ± 2.13	34.1 ± 4.7	**0.048**
(minimum; maximum)	(30; 34)	(28; 43)	
MRC score in left ED m. (mean ± SD)	4.1 ± 0.83	4.05 ± 0.73	0.85
MRC score in right ED m. (mean ± SD)	4.25 ± 0.88	3.89 ± 0.83	0.36

The bold values indicate statistically significant *p* values.

**Table 2:** Patient characteristics in different subgroups among the examined ALS patients. The *p* values were calculated between the subgroups within the rows. The statistical analysis is given in the ‘Materials and methods’

Onset symptoms	Bulbar	Right upper limb	Left upper limb	Right lower limb	Left lower limb	*p*
Number of patients	8	5	4	4	5	—
Number of female/male	5/3	2/3	1/3	2/2	1/4	0.56
Age (years) (mean ± SD)	57.5 ± 10.0	67 ± 11.81	69.75 ± 5.35	64.5 ± 15.79	70.4 ± 12.83	0.21
Disease duration *(from onset to referral, examination)*(months) (mean ± SD)	6.5 ± 1.2	5.4 ± 1.52	5.75 ± 1.26	7.25 ± 0.50	5.6 ± 1.67	0.19
ALSFRS-R (mean ± SD)	30.4 ± 2.13	35.8 ± 4.68	38.75 ± 2.87	31.5 ± 3.48	30.8 ± 2.17	**0.008**
MRC score in left ED m. (mean ± SD)	4.61 ± 0.83	4.4 ± 0.54	4.5 ± 0.57	4.25 ± 0.5	3.8 ± 0.45	0.4
MRC score in right ED m. (mean ± SD)	4.25 ± 0.88	4.2 ± 1.09	4.25 ± 0.5	3.25 ± 1	3.8 ± 0.84	0.29

The bold values indicate statistically significant *p* values.

Comparing subgroups and functional scales, significant differences were observed when comparing the ALSFRS-R scores among the subgroups (Table 1).

When comparing the LUL and RUL groups, we did not find a statistically significant difference. However, significant differences were observed when comparing the LUL group with all the other subgroups. Similarly, a significant difference was found between the RUL groups.

Based on these results, it is evident that patients in the LUL subgroup exhibited the mildest clinical symptoms, whereas those in the bulbar subgroup had the most severe symptoms.

We also compared the subgroups based on the MRC strength score on the ED muscle, both on the left and right sides. Differences were not significant among the groups if MRC scores in ED were compared (Tables 1 and 2).

### 3.2 SFEMG

The values obtained from the SFEMG examination of all ALS patients (*N* = 26) in the three examined muscles were compared with healthy controls (*N* = 26) (Table 3). Significant differences were observed in the mean jitter, mean percentage of increased jitter, FD, and blocking percentage (Table 3). In Table 3, we show a detailed summary of the parameters within the bulbar-onset and limb-onset groups; the only significant difference could be detected in ED muscle by the mean jitter and increased jitter percentage, but the sample numbers of the subgroups (e.g., 2) by the muscle alone are imbalanced and small which might result in misleading interpretations.

**Table 3:** SFEMG parameters among the examined ALS patients and healthy controls

ED muscle	Mean jitter (ms)	Increased jitter percentage	Block percentage	FD
Healthy controls (*N* = 26)	32.5 ± 2.8	0%	0%	1.3 ± 0.1
ALS patients (*N* = 26)	54.4 ± 4	30.9% ± 14.2%	15.4% ± 10.7	2.6 ± 0.45
***p*** **-value**	**<0.001**	**<0.001**	**<0.001**	**<0.001**
*Bulbar-onset* (*N* = 8)	56.74 ± 4.42	39.38 ± 16.69%	16.88 ± 11.93%	2.38 ± 0.35
*Limb-onset* (*N* = 18)	53.34 ± 3.4	27.11% ± 11.59%	14.72% ± 10.36%	2.66 ± 0.47
***p*** **-value**	**0.04**	**0.039**	0.64	0.15
**Orbicularis oculi muscle**				
Healthy controls (*N* = 26)	35.7 ± 2.8	0%	0%	1.2 ± 0.1
ALS patients (*N* = 7)	51.5 ± 3.0	23.6% ± 11.1%	15.7% ± 37	2.2 ± 0.23
***p*** **-value**	**<0.001**	**<0.001**	**<0.001**	**<0.001**
*Bulbar-onset* (*N* = 2)	53.4 ± 3.39	32.5 ± 10.61%	22.5 ± 17.68%	2.1 ± 0.28
*Limb-onset* (*N* = 5)	42.68 ± 13.86	26.67% ± 5.77%	13.0% ± 12.04%	2.2 ± 0.23
***p*** **-value**	0.35	0.37	0.43	0.64
**Frontalis muscle**				
Healthy controls (*N* = 26)	37.9 ± 2.6	0%	0%	1.3 ± 0.13
ALS patients (*N* = 8)	51.24 ± 2.4	23.75% ± 6.94%	23.13% ± 8%	2.14 ± 0.15
***p*** **-value**	**<0.001**	**<0.001**	**<0.001**	**<0.001**
*Bulbar-onset* (*N* = 4)	51.78 ± 1.16	26.25 ± 4.79%	27.5 ± 5.0%	2.18 ± 0.13
*Limb-onset* (*N* = 4)	45.7 ± 13.2	25.0% ± 5.0%	18.75% ± 8.54%	2.1 ± 0.18
***p*** **-value**	**0.39**	**0.73**	**0.13**	**0.5**

The bold values indicate statistically significant *p* values.

**Figure 1:**
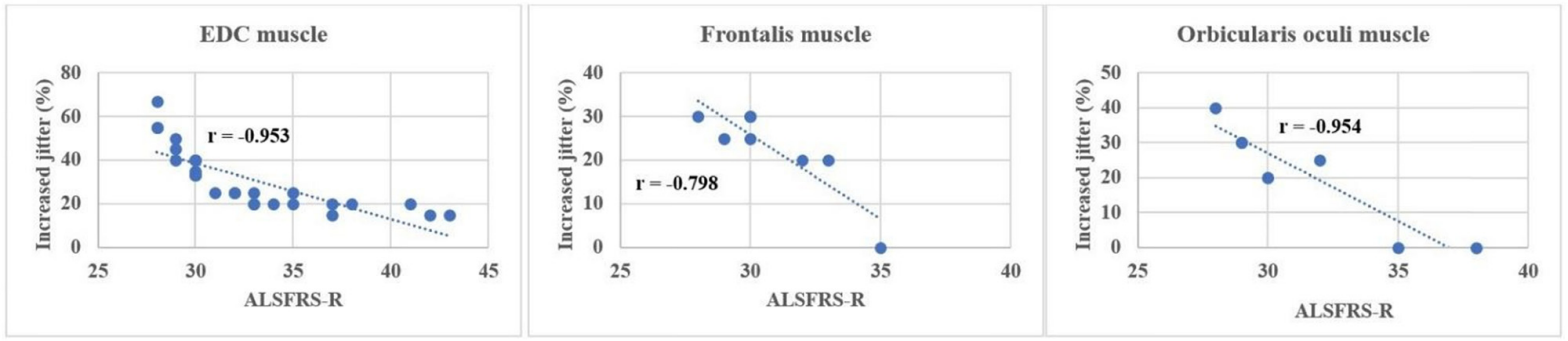
Correlation between ALSFRS-R and percentage of increased jitter in the examined muscles (ED m.: *p* < 0.0001, frontalis m.: *p* = 0.02, orbicularis oculi m. *p* < 0.0001).

**Figure 2:**
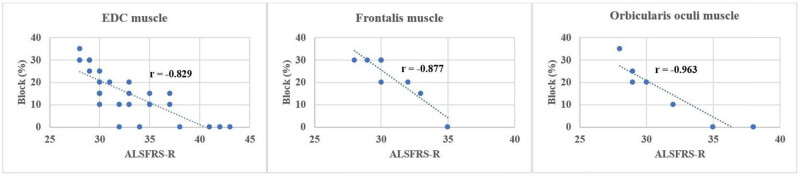
Correlation between ALSFRS-R and percentage of blocks in the examined muscles (ED m.: *p* < 0.0001, frontalis m.: *p* = 0.004, orbicularis oculi m.: *p* = 0.005).

**Figure 3:**
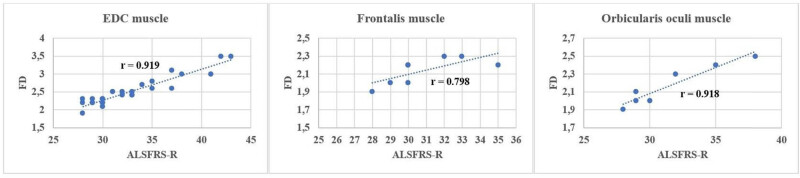
Correlation between ALSFRS-R and FD in the examined muscles (*p* < 0.0001 in all).

**Figure 4:**
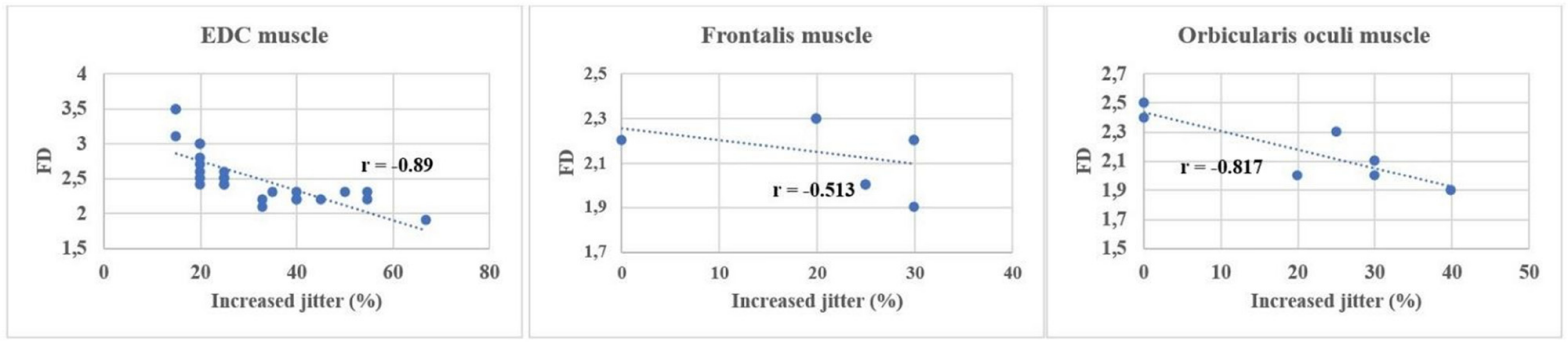
Correlation between FD and percentage of increased jitter in the examined muscles (ED m.: *p* < 0.0001, m. frontalis: *p* = 0.19, m. orbicularis oculi *p* = 0.03.

Comparing ALSFRS-R and the indices of the SFEMG examination of the ED muscle, a significant negative correlation (Table 4) was found in both the percentage of increased jitter (*r* = −0.953) (Figure 1) and blocking percentage (*r* = −0.829) (Figure 2) (*p* < 0.001 in all cases). FD was positively correlated with ALSFRS-R (*r* = 0,919; *p* < 0.001) (Figure 3). All results show a very strong correlation. A significant correlation was also detected between the percentage of increased jitters and FD (*r* = 0.89, *p* < 0.001) (Figure 4).

**Table 4:** Correlation between the clinical scales and neurophysiological parameters

	Parameter	Mean jitter	Increased jitter (%)	Block (%)	FD
ED m. all patients *N* = 26	ALSFRS-R	*r* = −0.901	*r* = −0.953	*r* = −0.829	*r* = 0.919
		*p* < 0.0001	*p* < 0.0001	*p* < 0.0001	*p* < 0.0001
	MRC in ED	*r* = −0.441	*r* = −0.469	*r* = −0.729	*r* = 0.53
	m.	*p* = 0.024	*p* = 0.02	*p* < 0.0001	*p* = 0.005
	Increased	—	—	—	*r* = −0.89
	jitter %				*p* < 0.0001
ED m. RUL group excluded *N* = 21	ALSFRS-R	*r* = −0.340	*r* = −0.96	*r* = −0.824	*r* = 0.901
		*p* = 0.09	*p* < 0.0001	*p* < 0.0001	*p* < 0.0001
	MRC in ED	*r* = −0.437	*r* = −0.464	*r* = −0.789	*r* = 0.42
	m.	*p* = 0.055	*p* = 0.03	*p* < 0.0001	*p* = 0.06
	Increased	—	—	—	*r* = −0.888
	Jitter %				*p* < 0.0001
Frontalis m. *N* = 8	ALSFRS-R	*r* = −0.805	*r* = −0.798	*r* = −0.877	*r* = 0.798
		*p* = 0.016	*p* = 0.02	*p* = 0.004	*p* = 0.02
	MRC in ED	*r* = −0.252	*r* = −0.007	*r* = 0.189	*r* = 0.425
	m.	*p* = 0.55	*p* = 0.99	*p* = 0.65	*p* = 0.29
	Increased	—	—	—	*r* = −0.513
	jitter %				*p* = 0.19
Orbicularis oculi m. *N* = 7	ALSFRS-R	*r* = −0.964	*r* = −0.954	*r* = −0.963	*r* = 0.918
		*p* = 0.0005	*p* < 0.0001	*p* = 0.0005	*p* = 0.004
	MRC in ED	*r* = −0.934	*r* = −0.864	*r* = −0.904	*r* = 0.187
	m.	*p* = 0.003	*p* = 0.01	*p* = 0.005	*p* = 0.02
	Increased	—	—	—	*r* = −0.817
	Jitter %				*p* = 0.03

The right ED muscle might have been affected and the SFEMG parameters might have been more pathological in the RUL group, so we wanted to perform an analysis excluding this subgroup, to see the alterations. When the RUL-onset group was excluded, the correlations were as follows: ALSFRSR compared to jitter percentage (*r* = −0.96), and block percentage (*r* = −0.824) (Table 4). A significant negative correlation was found in the following indices obtained from the SFEMG examination of the frontal muscle and between the ALSFRS-R: mean jitter *r* = −0.805; *p* < 0.01; mean percentage of increased jitter *r* = −0.798; *p* = 0.02 (Figure 1); and blocking percentage *r* = −0.877; *p* = 0.004 (Figure 2). The FD was positively correlated with ALSFRS-R (*r* = 0.798; *p* = 0.02) (Figure 3 and Table 4). With EMG examination no denervation, fasciculation or polyphasic potentials (indicating reinnervation) were detected among the factors that might influence the result of SFEMG. Therefore, the observed variations during the SFEMG examination are not due to EMG abnormalities.

**Figure 5:**
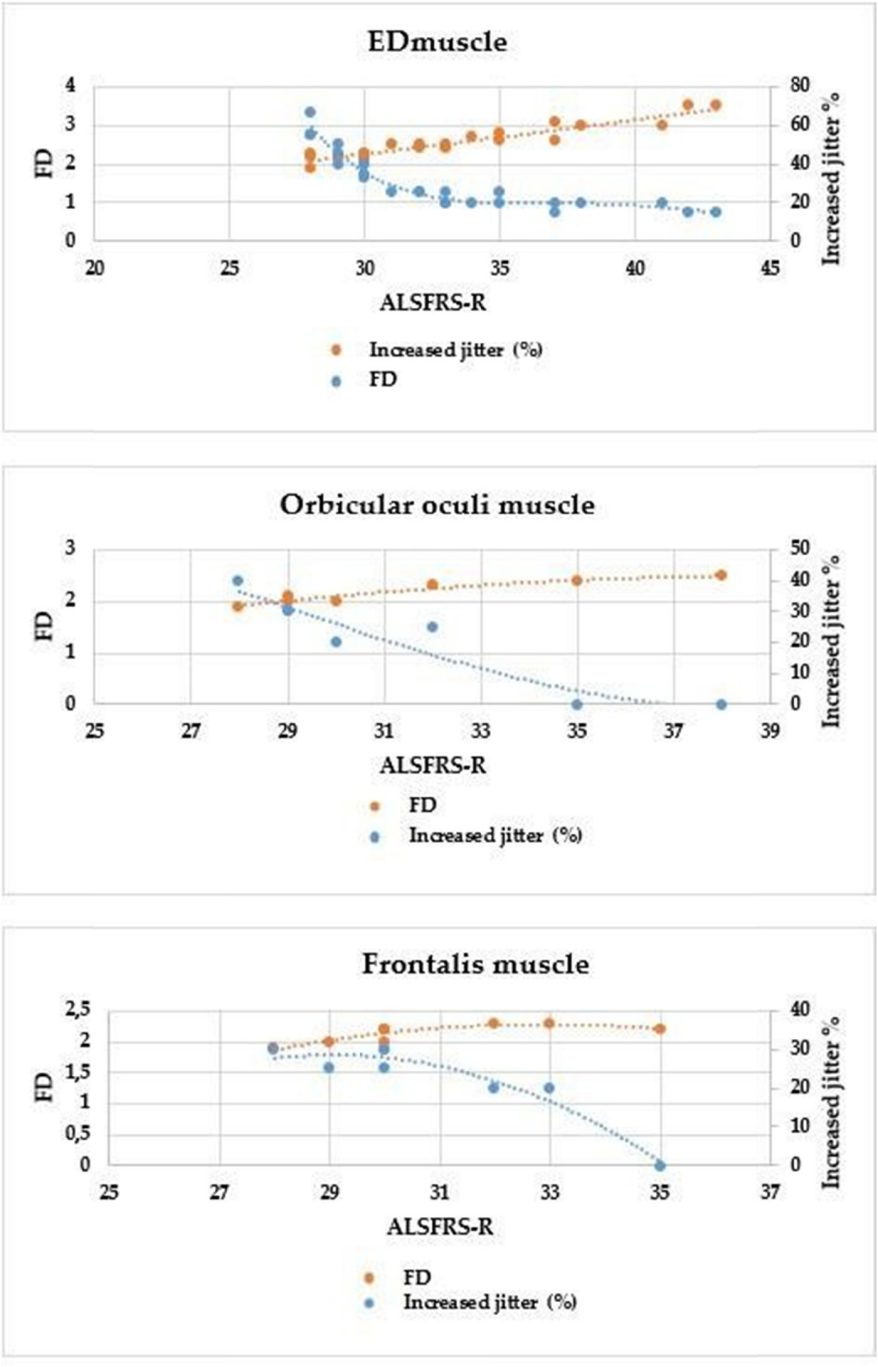
Correlation between ALSFRS-R, FD, and increased jitter % in ED muscle, frontalis muscle, and orbicularis oculi muscle.

Between the ALSFRS-R and the indices of the SFEMG examination of the orbicularis oculi muscle, a significant negative correlation was found in the mean jitter (*r* = −0.964), in the mean percentage of increased jitter *r* = −0.954 and blocking percentage *r* = −0.963 and, respectively, *p* < 0.001 in all cases. These results show a very strong correlation. Similarly, as in the case of the ED muscle and the frontal muscle, the FD was positively correlated with ALSFRS-R in this instance as well (*r* = 0,918; *p* < 0.01), resulting in a very strong correlation. Figure 5 shows the correlation between the percentage of increased jitter, FD, and ALSFRS-R. The percentage of increased jitter increases and FD decreases in more severe stages of the disease, marked by more severe ALSFRS-R scores. In all muscles, a notable value of ALSFRS-R can be detected: 31 points in the ED muscle, 30 in the orbicular oculi muscle, and 31 in the frontal muscle. This shift from around 30 points signifies the commencement of a process wherein reinnervation capacities are depleted, coinciding with a reduction in FD.

**Figure 6:**
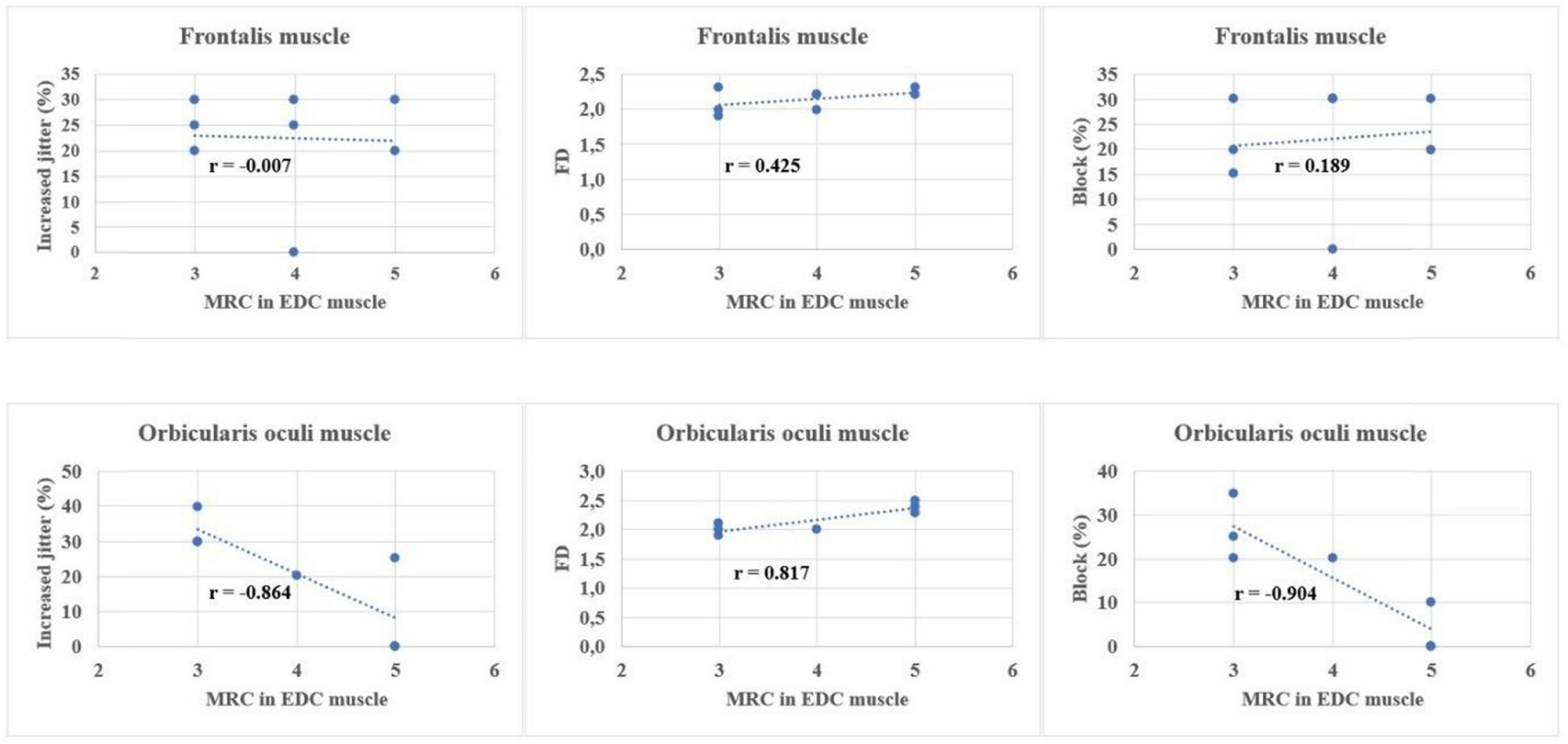
Correlation in frontal muscle and orbicularis oculi muscle between increased jitter percentage, FD and block percentage, and MRC score in ED muscle.

Since the examination of the ED muscle was performed on the right side for all patients, we compared their results with the right-sided MRC score of the ED (Table 4). ED muscle strength and the following three SFEMG indices showed significant negative correlation: mean jitter (*r* = −0.441; *p* = 0.02, moderate correlation) mean percentage of increased jitter (*r* = −0.469; *p* = 0.02 moderate correlation) and blocking percentage (*r* = −0.729; *p* < 0.001 strong correlation). Excluding the RUL-onset group from the analysis, similar results were detected (Table 4), although significance was not reached. In case of the orbicularis oculi muscle examining the correlations between the MRC score in the ED muscle with the percentage of jitter, blocks, and FD, we found a significant correlation in all respects (Table 4), but in the frontalis muscle only in case of jitter percentage. In the frontalis muscle, jitter percentage and FD did not show a significant correlation either (Figure 6, Table 4).

Disease duration did not show a significant correlation with ALSFRS-R in the examined cohort (Table 5).

**Table 5:** Correlation between ALSFRS-R in all patients and subgroups with disease duration

ALSFRS-R	All patients, *N* = 26	Bulbar-onset group, *N* = 8	Limb-onset group, *N* = 18
Disease duration	*r* = −0.34 *p* = 0.09	*r* = −0.422 *p* = 0.297	*r* = −0.28 *p* = 0.26

## 4 Discussion

Despite adequate information about case history and a comprehensive physical examination, many ALS patients are diagnosed only in the late stage. Our aim was to find such hallmarks with the help of SFEMG. These might help to optimize the supporting care of patients living with ALS and might also help in clinical trials. All these can lead to a better quality of life for a longer period of time, which is of great importance for patients and their families alike [3,15,25].

In our study, the only significant difference between the subgroups of bulbar-onset and limb-onset was mean age. The patients of the “bulbar” subgroup were significantly younger. Although some patients were already in a severe condition, the time between disease onset to referral did not differ much. The disease duration did not show a significant correlation with the functional status, which might be due to the sample size. But also, important to note, that the minimum–maximum time was 5–8 months, which is very narrow, and cannot differentiate well between rapid and less rapid progression. This is contrary to the findings in a Spanish study, where they found a correlation between the disease duration and SFEMG findings, but the time was much higher (21−13 months) [26]. Unfortunately, especially in four patients suffering from the bulbar type, the progression was very rapid. Also, as many patients were in a poor overall condition on arrival, we think that our study is important in early diagnosis. On the basis of the onset symptoms and time of onset – always established according to the history of the patient (auto and hetero) – the first symptoms might have appeared much earlier, but the disease was not recognized or it might have been ignored by the patient. Time is important in estimating survival: in a publication on a referral ALS series, the authors claim the progression of ALSFRS-R during the whole disease and the first 100 days after diagnosis is closely related to the length of time [25]. In our study, the worst ALSFRS-R scores were found in the bulbar group. Compared with the limb-onset group, the prognosis in the bulbar subgroup was worse, similar to those reported in a review by Kiernan [3]. MRC score in ED muscle did not show significant differences among the groups. Since ALS is usually asymmetric and the symptoms in the dominant limb might also be suggestive of a worse prognosis [27] RUL, RLL, LUL, and LLL, subgroups were created for comparison. Because our patients were right-handed, we could not create a dominant and non-dominant group. Nevertheless, there was no difference in the onset of symptoms between the two limb sides (Table 2). The longest time from onset to referral was found in the lower-limb onset group, but it was not significant; the symptoms were probably more alarming in the other subgroups. When ALSFRS-R results were compared among the subgroups, the worst data were seen in the two lower limb groups (LLL, RLL) and were similar to those in the bulbar-onset group. The slight difference between LUL and RUL might be explained by the dominance of the upper limb [27]. Devine et al. assume based on their investigations that limb dominance may be a significant factor underlying the onset and spread of ALS [27]. This finding might certainly be due to a small sample size. But, as we mentioned above, it might have an impact on the prognosis as well [27]. The correlation between SFEMG findings and functional status may contribute to the cautious implication that it might be useful even in estimating prognosis. According to our findings, we think that SFEMG might be useful in the neurophysiological arsenal similar to other studies [13,28,29]. Limb dominance may reflect underlying neuronal vulnerabilities [27]. According to another opinion, a poorer prognosis in lower limb-onset ALS might be correlated to the risk of thromboembolic disease and infections due to immobility [30]. By comparing MRC scores in ED muscle, it could be seen that if the right limb was affected the scores were lower, also showing the importance of sides in ALS [30].

Despite the fact that the facial muscles were clinically unaffected, compared to the healthy controls’ mean jitter, mean percentage of increased jitter, FD, and blocking percentage, significant differences were observed in all patients, aligning with findings from other studies in the field [28]. These parameters might be useful in the early stage of ALS or when the diagnosis is uncertain, e.g., because the patient had sensory neuropathy due to other reasons. Incorporating facial muscle SFEMG examination may be also logical, since we do not expect FD to be increased in the face in polyneuropathies. In the publication of Liu et al. remarkably increased jitter, impulse blocking with increased FD supports the diagnosis of ALS rather than cervical spondylosis [28]. In cases like that, SFEMG might be a supportive tool [29]. Comparing the SF EMG parameters detailed in the three muscles separately among the subgroups of bulbar- and limb-onset cannot be interpreted unequivocally because of the small sample size.

We also correlated the results of SFEMG studies with the ALSFRS-R and the MRC score in ED. Our results revealed a significant negative correlation between the functional status and comparison of mean jitter, percentage of increased jitter, and blocking percentage. Conversely, FD showed a significant positive correlation with the functional scales, indicating a gradual decrease in FD values as the disease progressed. We assume that SFEMG findings in a given patient at a specific time point may contribute to the estimation of the prognosis of the disease, since it correlates with the ALSFRS-R, which is a prognostic factor, with cautious interpretation [29,31,32]. Our findings were similar to those of Imam, who assumed that a higher FD value might be predictive of a better prognosis [29]. An increase in FD might suggest reinnervation before muscle biopsy findings would confirm it [8]. It might be interpreted also as a consequence of possible ephaptic activation of neighboring fibers resulting in increased FD. They do not necessarily appear as a fiber-type grouping in biopsies [8]. Therefore, we assume that FD and jitter have to always be analyzed together (Table 4 and Figure 5) because this way these two parameters together might serve as better prognostic factors. All correlations were significant in the ED and orbicularis oculi muscles. As for the frontalis muscle, the correlation was not significant, but the trend was similar. We have observed a notable value of ALSFRS-*R* value of 30 in the examined muscles, the percentage of increased jitter is high, and FD is already low. This delineates the initiation of a process where reinnervation capacities are exhausted, coupled with a decline in FD. The observed percentages of increased jitter further emphasize the significance of this point. Reinnervation at this stage is probably not so intense, already severe in instability in the neuromuscular junction can be detected because of the denervation, and the clinical score indicates a severe condition. In ALS according to animal studies, the remodeling of the neuromuscular junction is dynamic. A study by Dantes and McComas showed that the sizes of the motor unit potentials continued to increase as the disease progressed, and later the sprouting by surviving motor axons remained proportional to the numbers of denervated muscle fibers available for adoption [33] and the compensatory reinnervation may not last [33,34]. A possible tool for monitoring could also be motor unit size index, which measures a clinically relevant reinnervation effect [34].

These findings are interesting because our patients had no symptoms in any of their facial muscles. Contrary to limb muscles, facial muscles tend to be relatively spared during the early stages of the disease, so we were curious to investigate whether we could detect any differences in these muscles with SFEMG examinations. Axonal-stimulating SFEMG studies were performed in the frontalis muscle by Watanabe et al. and stated that in the relatively preserved frontalis muscle in ALS, jitter was significantly increased, possibly caused by dysfunction of the presynaptic neuromuscular junction and motor nerve terminals [35].

For this purpose, we conducted SFEMG examinations of the frontalis muscle and the orbicularis oculi muscle. In case of the frontalis muscle and orbicularis oris muscle, all parameters (Table 3) were within the reference values [22] but differed significantly from the data in healthy controls. However, it should be remembered that the low number of cases might contribute to these results. In our study when correlations concerning increased jitter percentage, FD, and block percentage and ALSFRS-R scores were examined, a significantly strong correlation was detected in both of the facial muscles, but when the parameters are correlated to MRC score in ED, it can be detected only in orbicularis oculi muscle (Figure 6, Table 4). Independently of the small sample size, the orbicularis oculi muscle appears more preferable than the frontal muscle to support early neurophysiological abnormality in a clinically unaffected muscle.

We assume that the findings in the facial muscles are important, although the sample is size small. Contrary to limb muscles, the cause for spared extraocular muscles is the difference in the expression of Wnts, described in a publication by [36]. The family of Wnt proteins plays a role in neuromuscular development and regeneration in extraocular and limb muscles, particularly at the level of neuromuscular junctions [36]. The study suggested that these proteins played a role in the pathophysiology of ALS [36]. Their data supported the presence of preserved Wnts in the neuromuscular junctions of extraocular muscles [36,37]. Nevertheless, in later stages, a possible dying back mechanism might be responsible for the lesion of the extraocular muscles. Microinjury of Piezo2 on muscle spindle proprioceptive terminals is supposed to be the underlying pathomechanism [38]. Our patients were examined in an early stage (6 months from symptom-onset), and their facial muscles were clinically preserved. Using a diagnostic tool specialized for neuromuscular junction abnormalities, we found pathological responses and strong correlations with ALSFRS-R and MRC scores in ED. This is the level where Wnt proteins are expressed [36]. Our findings suggest that with the help of combined jitter and FD analysis, the pathological changes can be detected before the appearance of clinical symptoms. We also assume that the orbicularis oculi muscle might be the best choice to support an early neurophysiological abnormality among clinically unaffected muscles.

## 5 Limitations

We are aware of the limitations of the study. Regarding research on SFEMG in ALS patients, only a relatively small number of patients have been involved [28,29] which limits the generalizability of the results. Unfortunately, when performing subgroup analysis, the sample size became smaller, which contributed to a weaker statistical power. Due to the real-life scenario there was an imbalance between right and left handedness, all patients enrolled were right-handed by chance. Nevertheless, every additional study result, even with a small number of participants, can significantly contribute to a more precise understanding of the role of SFEMG in the diagnosis of ALS. Our studies encompassed not only neurophysiological findings but also results of physical examination. We encountered challenges during the studied time interval due to the COVID-19 pandemic, which made it difficult to diagnose potential patients and access and examine those already diagnosed with ALS, while also disrupting our research processes. Although many patients had already severe symptoms, this also reflects the real-life scenario, at the time of the verified diagnosis the patients already have severe symptoms. However, it is important to note that all these patients were examined within a single center and region, resulting in a homogeneous study population. Nevertheless, other SFEMG studies in ALS encompass similar study population [26,28,29,35]. Given the rarity of the disease and the limited number of cases, it is essential to follow up with patients for an extended period and expand the study to include a larger sample of patients diagnosed with ALS.

## 6 Conclusion

Based on our results, we can assert that – in line with previous literature – SFEMG examination is a valuable tool in better understanding ALS and might be a potential tool in the future for assessing prognosis. According to our experience, a combined analysis of data jitter and FD gives the best correlation with clinical scales, and besides the ED muscle, the orbicularis oculi muscle is also important in the assessment.
